# Effect of sex and autism spectrum disorder on oxytocin receptor binding and mRNA expression in the dopaminergic pars compacta of the human substantia nigra

**DOI:** 10.1098/rstb.2021.0118

**Published:** 2022-08-29

**Authors:** Sage S. Frehner, Kip T. Dooley, Michelle C. Palumbo, Aaron L. Smith, Mark M. Goodman, Karen L. Bales, Sara M. Freeman

**Affiliations:** ^1^ Department of Biology, Utah State University, Logan, UT 84322, USA; ^2^ California National Primate Research Center, University of California Davis, Davis, CA 95616, USA; ^3^ Department of Behavioral Neuroscience, Oregon Health Sciences University, Portland, OR 97239, USA; ^4^ Department of Radiology, Emory University, Atlanta, GA 30322, USA

**Keywords:** autism, dopamine, oxytocin, substantia nigra, tyrosine hydroxylase

## Abstract

Oxytocin is an endogenous neuropeptide hormone that influences social behaviour and bonding in mammals. Variations in oxytocin receptor (OXTR) expression may play a role in the social deficits seen in autism spectrum disorder. Previous studies from our laboratory found a dense population of OXTR in the human substantia nigra (SN), a basal ganglia structure in the midbrain that is important in both movement and reward pathways. Here, we explore whether differences in OXTR can be identified in the dopaminergic SN pars compacta of individuals with autism. Postmortem human brain tissue specimens were processed for OXTR autoradiography from four groups: males with autism, females with autism, typically developing (TD) males and TD females. We found that females with autism had significantly lower levels of OXTR than the other groups. To examine potential gene expression differences, we performed *in situ* hybridization in adjacent slides to visualize and quantify OXTR mRNA as well as mRNA for tyrosine hydroxylase. We found no differences in mRNA levels for either gene across the four groups. These results suggest that a dysregulation in local OXTR protein translation or increased OXTR internalization/recycling may contribute to the differences in social symptoms seen in females with autism.

This article is part of the theme issue ‘Interplays between oxytocin and other neuromodulators in shaping complex social behaviours’.

## 1. Introduction

Autism spectrum disorder (ASD) is a prevalent developmental disorder that affects up to 1 in 55 children globally [1,2]. Symptoms of ASD include delayed learning, communication deficiencies, behavioural issues and acute anxiety [2–4]. The deficits in social interactions specifically can create unique challenges for some individuals with ASD, as well as for their family members and peers. Finding a treatment to manage or reduce the social symptoms of ASD has been a goal for numerous researchers looking to increase the quality of life for individuals with ASD over the past few decades.

Intranasal oxytocin (OT) is an experimental treatment option for individuals with ASD that has been the subject of many years of recent research. OT is a neuropeptide hormone produced in the hypothalamus which was first recognized to stimulate uterine contractions during labour and facilitate lactation. Beyond its role in female reproductive physiology, OT has also been acknowledged to act as an important pro-social hormone in both males and females [5,6]. Clinical trials administering intranasal OT to individuals with ASD have reported significant symptom improvements and increased sociability [7–9]. However, the neurobiological mechanisms by which OT acts to produce these results in humans is still not completely understood, and several papers have now demonstrated that OT's impact on social behaviour is not consistently positive and depends on the context and the individual's developmental experience, sex and genetic background [10–15]. Therefore, it is even more imperative that we investigate the fundamental neuroanatomy of the oxytocin system in the human brain in order to better understand the variable ways in which OT can modulate social behaviour.

Most of what we know about the behavioural effects of OT comes from research in rodent models. Monogamous species, such as the prairie vole (*Microtus ochrogaster*), have higher levels of oxytocin receptors (OXTR) than closely related non-monogamous rodents from the same genus [6]. Infusions of OXTR antagonists into various behaviourally relevant brain regions can block the formation of a pair bond in prairie voles [6,16] and can even inhibit maternal care towards pups [17]. These effects can be reversed by the infusion of OT or an OXTR agonist [17,18]. For example, when OT is released into the ventral tegmental area (VTA), which is a key component of the brains' reward system, social behaviours are promoted and increase in frequency [16].

The VTA is one of the primary producers of dopamine (DA) in the central nervous system, with the remainder of DA being produced in the substantia nigra (SN) pars compacta (SNc) [19,20]. The VTA projects to both the mesocortical and mesolimbic pathways, which in turn enables cognitive and emotional processes to influence behaviours tied to motivation and addiction [6,21]. The DA neurons in the VTA have been found to contain high levels of OXTR in mice, perhaps explaining why OT acting in the VTA promotes social behaviours and influences the saliency of social stimuli [22]. In fact, infusion of OT into the VTA causes DA to be released in downstream brain regions such as the nucleus accumbens and the medial prefrontal cortex [17,18].

The DA hypothesis of autism [2] suggests that the behavioural deficits seen in ASD arise from a dysfunction of midbrain dopaminergic systems, such as those of the mesocortical and mesolimbic pathways. Along with the multitude of rodent studies implicating the involvement of OT to activate DA in these pathways [17,18,22–25], human studies using fMRI and plasma extractions have offered additional confirmation [5,26]. It is currently believed that OT can block defensive and aggressive behaviours while linking this behavioural inhibition with the activation of the DA pathways to create and promote rewarding social behaviours [25]. With the interactions of OT on social reward already being well established in regards to projections from the VTA, the question still remains on how—and if—OT regulates the other DA-producing area: the SNc.

The SN is part of the basal ganglia—a brain region largely responsible for motor movements but also a primary component of the mesolimbic pathway. The SN can be divided into two distinct compartments, the SN pars-reticulata (SNr), which predominantly contains inhibitory GABA-ergic neurons, and the SNc, which contains the cell bodies of dopaminergic neurons [27]. It is plausible that OT could be acting upon the DA neurons in the SNc, along with those in the VTA, to modulate the saliency of social stimuli. However, unlike the stimulatory effects on DA neurons seen in the VTA, OT inhibits DA neurons in the SNc of mice [24]. Because the SNc and the rest of the basal ganglia mainly act to produce motor movements, OT in the SNc may be contributing to motor inhibition that is required for some types of top-down executive functions. The performance of many executive functions such as emotional processing and decision making during social situations have been connected to motor inhibition in healthy individuals [28]. These same executive functions have been found to be impaired in people with autism [29,30], with the most severe impairments being reported in diagnosed females [31]. However, ASD females tend to camouflage many of these symptoms either through internalization or by masking, in which observed social behaviours are mirrored [32–34]. Due to this camouflaging effect, females often take longer to be diagnosed than males or they may go their entire lives without being diagnosed [33,34]. The internalization of symptoms often leads to more overall distress and a higher likelihood of comorbidities such as depression or anxiety among females with ASD [32].

Sex differences in the endogenous OT system may contribute to the variability seen in ASD symptom severity between the sexes. Circulating levels of OT have been reported to be higher in females than in males, which has been observed in neurotypical humans and rodent species alike [18,25]. It has been proposed that the elevated levels of OT in females may reduce some of the symptoms of ASD, contributing to why females tend to have less outwardly noticeable symptoms than males [25]. The current study is the first to evaluate whether OXTR differs in the SN of individuals with ASD compared to matched typically developing (TD) control specimens and whether sex differences exist in OXTR density in these populations.

## 2. Methods

### (a) Specimens and tissue preparation

**Figure 3.**
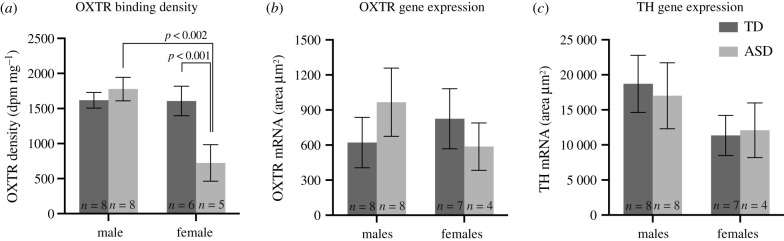
Effect of sex and ASD on receptor binding density and gene expression in the human SN. (*a*) OXTR binding density for TD and ASD males and females. Females diagnosed with ASD have significantly less OXTR binding than any of the other experimental groups. (*b*). OXTR mRNA within the SNc for TD and ASD males and females. No significant differences were identified between groups. (*c*) TH mRNA within the SN for TD and ASD males and females. No significant correlations were identified.

Unfixed, frozen blocks of de-identified, postmortem human brain tissue containing the SN of the midbrain were provided by the University of Maryland Brain and Tissue Bank, which is a brain and tissue repository of the NIH NeuroBioBank. The provided tissue includes 30 age-matched specimens in four distinct groups: TD males (*n* = 8), TD females (*n* = 7), males with ASD (*n* = 8) and females with ASD (*n* = 7). Due to some issues with tissue integrity during specimen processing, some specimens were not quantifiable (final sample sizes are reported for each outcome measure in figure 3). Age, race, cause of death and postmortem interval (PMI) information is provided in tables 1–4. The details of this study were reviewed by the Institutional Review Board at Utah State University and were found not to meet the criteria for human subjects research. The specimens were stored at −80°C, brought to −20°C, sectioned at 20 µm on a cryostat, and mounted to Fisher Superfrost-Plus slides. Slides were sealed in a box with a desiccant packet and stored at −80°C until use in receptor autoradiography, Nissl staining or *in situ* hybridization.

**Table 1.** Age in years.

	mean age ± s.d.	age range	median age
TD female	14.94 ± 8.27	4–27.87	16
TD male	17.43 ± 7.50	5.66–27.55	19.24
ASD female	15.09 ± 8.14	4.45–27.84	15.89
ASD male	17.25 ± 7.68	4.50–27	19.68

**Table 2.** Postmortem interval (PMI) in hours.

	mean PMI ± s.d.	PMI range	median PMI
TD female	19.71 ± 6.63	10–30	21
TD male	16.13 ± 5.51	10–24	15
ASD female	19.29 ± 9.59	5–34	22
ASD male	26.5 ± 19.15	3–63	19.5

**Table 3.** Cause of death.

	# TD specimens	# ASD specimens
smoke inhalation	1	1
multiple injuries	0	1
cardiac arrhythmia/cardiovascular disease/ dilated cardiomegaly	4	2
seizure/epilepsy related	0	3
drowning	1	2
cancer, complications of	0	1
subdural haemorrhage	0	1
suicide	2	1
struck by a car	0	1
peritonitis	0	1
respiratory failure/asphyxia	2	0
asthma	2	0
pneumonia/pseudomonas bronchopneumonia	2	0
gunshot wound to the chest	1	0
pending	0	1

**Table 4.** Race.

	# TD specimens	# ASD specimens
Caucasian	8	12
African American	7	3

### (b) Nissl staining to identify dopaminergic neurons

Based on previous work [35], we know that dopaminergic neurons in the SNc can be distinguished from the GABA-ergic neurons of the SNr when stained for Nissl substance using thionin. Fresh frozen 20 µm brain sections mounted to Fisher SuperFrost-Plus slides were kept in 4% paraformaldehyde at 4°C for 1 week in order to fix the tissue. Slides were then dipped in deionized water and soaked twice in 50/50% chloroform/ethyl alcohol for 1.5 h before being hydrated in descending concentrations of ethyl alcohol. The slides were dipped in 0.25% thionin, then water, before being dehydrated in ascending concentrations of ethyl alcohol and xylenes. Slides were coverslipped using CytoSeal 60 (Radnor, Wayne, PA, USA). The stained slides were examined using bright-field microscopy using a Keyence BZ-X800 (Keyence Corporation of America, Itasca, IL, USA) microscope. High-resolution images of the Nissl-stained sections were used to anatomically determine the boundaries of the pars compacta and thus inform the analysis of the OXTR autoradiograms.

### (c) Competitive-binding receptor autoradiography

Competitive-binding receptor autoradiography was performed to selectively reveal OXTR as previously described [36]. Until recently, locations of OXTR and the vasopressin 1a receptor (AVPR1a), a structurally similar and functionally related receptor, were not able to be dependably mapped with the use of the commercially available radioligands alone: ^125^I-ornithine vasotocin analogue (^125^I-OVTA) for OXTR and ^125^I-linearized vasopressin antagonist (^125^I-LVA) for AVPR1a (Perkin Elmer, Waltham, MA, USA). Because of the structural similarities between OXTR and AVPR1a, there is pharmacological cross-reactivity in this system [37]. When used in primate brains, these two radioligands are now known to bind to both receptors. In order to address the receptor cross-reactivity, we used a previously optimized method for visualizing OXTR and AVPR1a in the primate brain through the use of a modified form of receptor autoradiography, where the radioligand is co-incubated on the tissue with a selective competitor that blocks one of the receptor subtypes to reveal binding only to the receptor of interest. This approach has been validated in postmortem brain tissue from non-human primates [38–40] and humans [36,41,42] to selectively visualize either OXTR or AVPR1a. In the current study, we used this method of competitive-binding receptor autoradiography to specifically locate OXTR binding in the SN of the human brain.

Briefly, this approach involved first lightly fixing the sections in 0.1% paraformaldehyde (PFA; pH 7.4) followed by two washes in Tris buffer. Next, sets of three adjacent slides were incubated with ^125^I-OVTA for 1 h in one of three binding conditions: (i) 90 pM radioligand alone, (ii) 90 pM radioligand plus 10 nM SR49059 (Tocris, Minneapolis, MN, USA), a human-selective AVPR1a antagonist or (iii) 90 pM radioligand plus 100 nM ALS-II-69 (donated by ALS; see Smith *et al*. [43]), a human-selective OXTR antagonist. Accordingly, set (i) could be compared to sets (ii) and (iii) to confirm selective OXTR binding. After incubation, we washed the slides with Tris buffer, dipped them in ddH_2_O and air dried them. Last, the slides were exposed to Carestream BioMax MR film (Kodak, Rochester, NY, USA) for 10 days and then developed.

### (d) Quantification and analysis of autoradiograms

**Figure 1.**
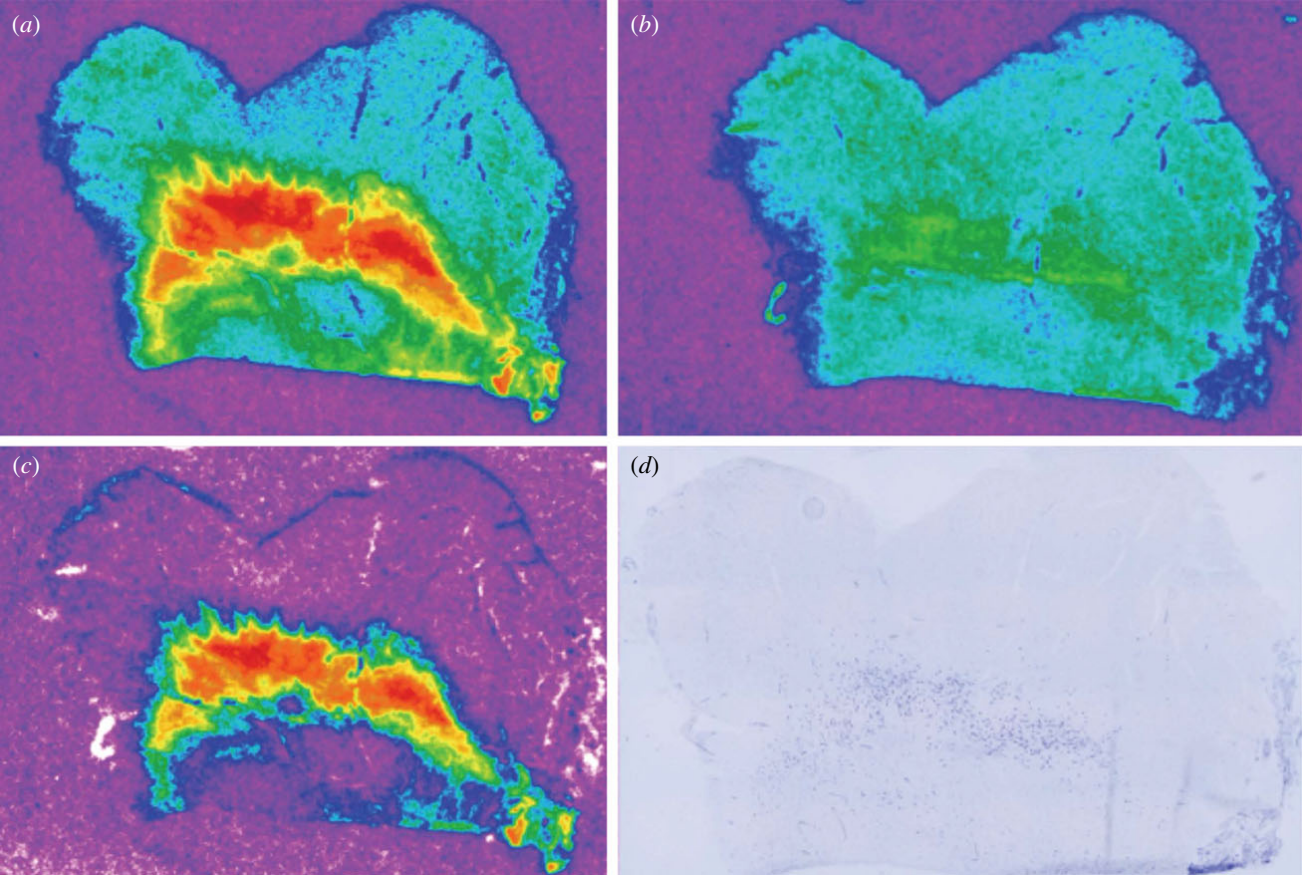
Total, nonspecific and specific OXTR binding in the human SN. (*a*) Total ^125^I-OVTA binding. (*b*) ^125^I-OVTA binding in the presence of 100 nM of OXTR antagonist ALS-II-69. (*c*) Digital subtraction of (*b*) from (*a*) to yield specific OXTR binding. (*d*) Nissl-stained tissue section showing the large, dopaminergic neurons of the pars compacta.

Three representative sections of the SN from each specimen were quantified. For each section, images of radioligand binding remaining in the presence of the AVPR1a competitor (i.e. nonspecific binding) were aligned to and digitally subtracted from the corresponding image of total radioligand binding to yield an image that represented specific OXTR binding (figure 1*a–c*). Digital densitometry was performed on the specific OXTR autoradiograms using MCID Core to quantify the density of OXTR in the SNc. Images of Nissl-stained tissue sections were placed side by side with the corresponding OXTR-specific binding autoradiogram in order to accurately outline the pars compacta on the autoradiogram (figure 1*d*).

### (e) *In situ* hybridization

**Figure 2.**
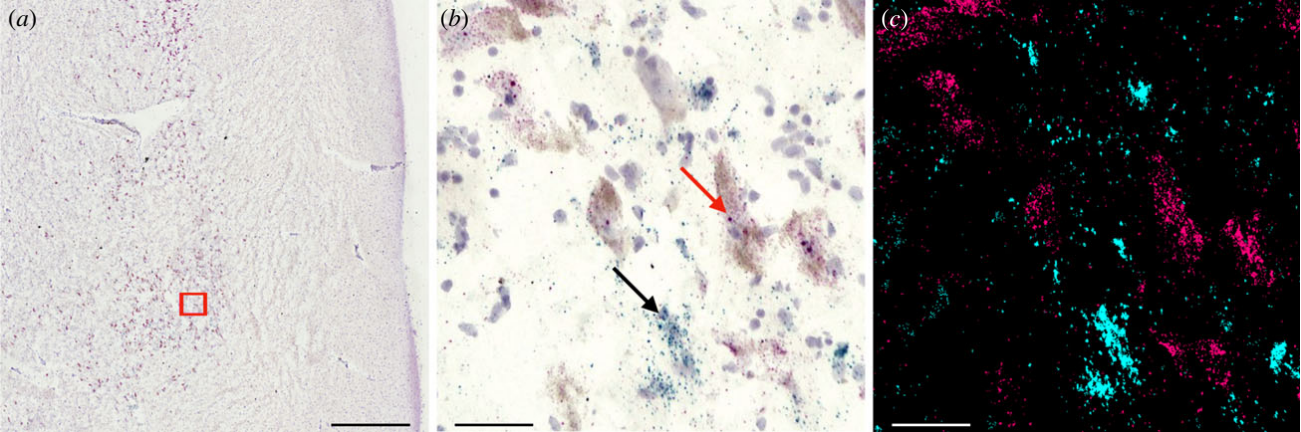
OXTR and TH mRNA expression in the human SN. (*a*) 4× microscope image of human SN tissue slice. Red box indicates location of (*b*) and (*c*). (*b*) 20× microscope image zoomed in from slice shown in (*a*). Red TH signalling indicated by red arrow; green OXTR indicated by black arrow. Cell nuclei appear purple, and the naturally occurring brown neuromelanin that is present in the dopaminergic neurons is also visible. (*c*) Macro created from image (*b*) for quantification; pink pixels represent TH mRNA and blue pixels represent OXTR mRNA. Scale bar of (*a*) is 750 µm, and scale bars of (*b*) and (*c*) are 50 µm.

We used RNAscope (ACD Bio, Inc) to visualize the mRNA of OXTR and tyrosine hydroxylase (TH) in the SN. TH is the rate-limiting enzyme found within dopaminergic neurons that catalyses the synthesis of DA from tyrosine. Dopaminergic neurons can be identified by the presence of condensed TH [44]. RNAscope is a highly sensitive method for *in situ* hybridization (ISH) which uses selective probes designed to bind to the RNA transcripts encoding desired proteins while simultaneously maintaining background suppression and conserving tissue morphology [45]. This method of ISH makes it possible to visualize multiple target genes within a single tissue sample by assigning different chromogenic labels to each target probe. Single mRNA molecules that are tagged in the tissue appear as coloured dots; in the current study, TH mRNA appears red/magenta, and OXTR mRNA appears green/cyan (figure 2*b*).

ISH was performed over a 3-day period with the tissue-mounted slides first being fixed overnight in 4% PFA (pH 7.4). On day 2, slides were removed from the PFA, rinsed and dehydrated in 50%, 70%, then twice in fresh 100% ethanol for 5 min each at room temperature. Following ethanol incubation, tissue was covered in hydrogen peroxide for 10 min, then rinsed twice in fresh distilled water. Next, slides were loaded into a vertical slide rack and submerged into boiling 1× Target Retrieval solution for 10 min with temperature being maintained between 98 and 102°C. Once removed, slides were again rinsed in fresh distilled water followed by a 3 min wash in 100% ethanol. After drying, individual tissue slices on each slide were surrounded by a hydrophobic barrier using an RNAscope ImmEdge pen. The tissue was then covered in Protease Plus and incubated for 30 min at 40°C. After 30 min, the slides were rinsed twice in fresh distilled water and the tissue samples were covered with channel probes. Each specimen was processed in triplicate sets of adjacent sections: one section was incubated in experimental probes (a combination of TH and OXTR probes), one section was incubated in the negative control probe (*dapb*, bacterial genes) and one section was incubated in the positive control probes (*PPIB* and *POL2RA*, two mammalian housekeeping genes; see electronic supplementary material, figure S1). The probes were allowed to hybridize for 2 h at 40°C. Following probe hybridization, slides went through two 2 min rinses in wash buffer and were then left overnight in 5× saline sodium citrate. On day 3, slides were processed through the 10 RNAscope amplification steps, one at a time, as well as the red and green chromogenic probe labelling steps. Cell nuclei were then counterstained by submerging the slides in 50% Gill's Hematoxylin-1 for 30 s. Finally, the slides were rinsed in fresh distilled water, dried at 60°C for 15 min, cooled and coverslipped using VectaMount (Vector Laboratories, Burlingame, CA, USA).

### (f) Quantification and analysis of *in situ* hybridization

Five representative images taken at 20× using a Keyence BZ-X800 bright-field microscope (Keyence Corporation of America, Itasca, IL, USA) were quantified from each specimen. Representative images were selected surrounding the dopaminergic cells expressing the strongest TH signalling inside of the SN (figure 2*a*). Microscope settings were maintained to an aperture of 100%, 75% transmitted light and a brightness/exposure of 1/120 s with edge-embossment set to ‘middle’ for each captured image. Our quantification approach used the hue of each of the chromogenic stains (red/magenta and green/cyan). Masks of each image were manually created using Keyence Corporation's BZ-X800 Analyzer (v. 1.1.2.4). The bright-field double extraction hybrid cell count function from this software was used with the first and second extractions being selected as the red-hue ISH stain from TH and the green-hue stain from OXTR, respectively (figure 2*c*). Each hue-based mask is made by incrementally adding the hues of interest by selecting individual pixels on one representative image and toggling the sensitivity and tolerance of the mask settings until the pattern of quantified pixel area aligns with true pattern of that coloured stain on the image being quantified. Those settings can then be saved as a macro and applied to all images in order to standardize the quantification approach across images. All of the resulting masks generated by the saved macro are visually checked to ensure that the quantified pixel area aligns with the visual distribution of signal stain. Signal intensity differences are taken into account by including lighter shades of the stain's hue as well as darker shades when creating the masks, so that the total pixel area covered by that hue's intensity range is included in our data. The resulting pixel area occupied by each probe was averaged over the five images per specimen.

In many cases, the mRNA transcripts were so prevalent that the stained dots appeared as clusters; for this reason, we opted not to use a dot-counting algorithm to quantify gene expression. We also did not directly perform a colocalization analysis, due to the challenges in accurately identifying the variable, mixed hue that results when two chromogenic stains appear on top of one another. We recognize the limitation in our approach here, which could have been avoided with the use of fluorescently labelled probes instead of chromogenic ones; however, the use of fluorescent tags in postmortem human brain tissue causes complex issues with accurate quantification due to the high prevalence of auto-fluorescing lipofuscin [46–48]. Therefore, we opted for a chromogenic labelling approach instead.

### (g) Statistical analysis

Statistical analyses and data visualization were performed in Graphpad Prism. Two-way ANOVAs were used to determine whether there was a main effect of ASD or sex on OXTR density, OXTR mRNA and TH mRNA in the SNc and to identify potential interaction effects between these two factors on our outcome measures. Linear regressions were used to evaluate whether there was a correlation between OXTR binding and age and between OXTR binding and PMI, with no significant correlations being identified (electronic supplementary material, figures S2 and S3, respectively). An exploratory linear regression analysis on a subset of ASD specimens was used to evaluate the potential relationship between OXTR density and ASD symptom severity by correlating OXTR binding with scores from the revised autism diagnostic inventory [49], although no significant correlations were identified (electronic supplementary material, figure S4).

## 3. Results

We found a main effect of sex (*F*_1,23_ = 8.461; *p* < 0.01; Hedges' *g* = 0.862) and an interaction effect between sex and diagnosis (*F*_1,23_ = 8.146; *p* < 0.01) on OXTR-binding levels in the SNc (figure 3*a*). The main effect of diagnosis trended toward, but did not reach, significance (*F*_1,23_ = 3.926; *p* = 0.0596; Hedges’ *g* = 0.480). A Šídák's *post hoc* test for multiple comparisons revealed significant differences between females with ASD and males with ASD (adjusted *p* = 0.0012; Hedges' *g* = 1.913) and between females with ASD and TD control females (adjusted *p* = 0.0093; Hedges’ *g* = 1.618). Females with ASD have less OXTR binding than the other groups, suggesting that they have fewer mature, cell surface OXTR located in the SNc.

This effect was not replicated in the mRNA measures obtained through ISH. There was no significant main effect of sex on OXTR mRNA (*F*_1,23_ = 0.1039; *p* = 0.7501) nor a main effect of diagnosis on OXTR mRNA (*F*_1,23_ = 0.03793; *p* = 0.8473), and no interaction effects (figure 3*b*). Similarly, we found no significant main effects or interactions in our analysis of TH mRNA levels between experimental groups (sex: *p* = 0.1708, diagnosis: *p* = 0.9114) (figure 3*c*).

## 4. Discussion

The current study reports a significantly lower density of OXTR in the SNc in females with ASD compared to unaffected females, unaffected males and males with ASD. This effect was not recapitulated at the level of gene expression; there were no significant differences between groups in the amount of OXTR mRNA present in the same region. This effect does not appear to be driven by overt, underlying differences in the dopaminergic system of the SNc, as there were no differences detected between groups in the amounts of mRNA for TH, the rate-limiting enzyme in the synthesis of DA. Although it needs experimental support from future studies, these results imply potential sex differences in ASD in the cellular turnover of mature, cell-surface OXTR, either in the internalization/desensitization of OXTR or in the local translation and insertion of new OXTR into neuronal membranes.

To our knowledge, this is the first study to report sex differences in OXTR binding in any region of the autistic brain. A previous study from our group characterized OXTR binding in five regions of interest in postmortem ASD brain tissue and found effects of ASD on OXTR-binding densities in two regions: the nucleus basalis of Meynert and the ventral pallidum [36]. However, no significant effects of sex nor interactions between sex and diagnoses were found, and no experiments were performed to quantify OXTR mRNA in those areas. Taken together, these two studies suggest regional differences in the regulation of OXTR levels in the human brain, which could contribute to the expression of species-typical social behaviour, such as an ASD phenotype.

Evidence from cell culture assays shows that OXTR are internalized via endocytosis after agonist binding, but are not degraded by lysosomes and return to the cell surface 4 h later [50]. Experimental follow-up assays from this same study demonstrated that receptor recycling, not de novo receptor synthesis, was responsible. Thus, if mRNA levels of OXTR do not differ across males and females with and without ASD, then the reduced levels of OXTR binding in females with ASD could be due to enhanced OXTR desensitization or delayed receptor recycling to the cell surface. Alternatively, it is possible that the SNc OXTR in females with ASD experience prolonged or continuous agonism, which, like other G protein-coupled receptors, would cause internalization and desensitization [50]. Because internalization of OXTR has been shown to be dependent on clathrin-dependent endocytosis [51], it is also possible that a broader issue with the endocytotic cycle may be at play. Follow-up studies should investigate these and other cellular targets of the endocytotic pathway in order to determine whether there are group differences that may support the results of the current study.

The reduced density of OXTR in the SNc of females with ASD might be contributing to the greater impairment in executive functioning that is experienced in females versus males with the disorder. This effect could be due to a deficiency in OT facilitating motor inhibition required for these types of advanced emotional and behavioural functions [24,28]. The heightened social competency displayed by ASD females marked by symptom masking and camouflaging may be influenced by the pro-social effects of endogenous OT in other brain areas. Due to the evident regional differences in protein and gene expression, future research should address expanding upon comparing OXTR-binding densities and mRNA among ASD and neurotypical samples of both sexes in other prominent social reward areas such as the VTA.

A majority of research on OXTR in postmortem brain tissue has focused on protein levels (i.e. receptor binding) only or mRNA quantification only, primarily through the use of receptor autoradiography or quantitative PCR, respectively. Antibody-based methods to quantify OXTR, such as immunohistochemistry and western blotting, have been unreliable due to issues with antibody specificity [36], leaving autoradiography and ISH as the most trusted options available, especially when working with non-mouse tissues. By using approaches that permit duplex or multiplex visualization of more than one target, we become capable of better characterizing the underlying neuronal circuits responsible for the action of OT. Furthermore, the discrepancies reported here between receptor density and mRNA levels suggest that future research should incorporate outcome measures at both levels of analysis, rather than relying on only one or the other in order to draw functional conclusions on the action of OT in the brain.

## Data Availability

The data are provided in the electronic supplementary material [52].
